# Age-Specific Trends in Morbidity, Mortality and Case-Fatality from Cardiovascular Disease, Myocardial Infarction and Stroke in Advanced Age: Evaluation in the Swedish Population

**DOI:** 10.1371/journal.pone.0064928

**Published:** 2013-05-31

**Authors:** Karin Modig, Tomas Andersson, Sven Drefahl, Anders Ahlbom

**Affiliations:** 1 Institute of Environmental Medicine, Karolinska Institute, Stockholm, Sweden; 2 Centre for Occupational and Environmental Medicine, Stockholm County Council, Stockholm, Sweden; 3 Department of Sociology, Demography Unit, Stockholm University, Stockholm, Sweden; Wayne State University School of Medicine, United States of America

## Abstract

**Background:**

It is not clear if the downward trend in cardiovascular disease (CVD) observed for ages up to 85 years can be extended to the oldest old, those 85 years and above.

**Methods and Findings:**

This nationwide cohort study presents age specific trends of CVD as well as for myocardial infarction (MI) and stroke separately for the period 1994 to 2010 for individuals 85 to 99 years old in Sweden. Data were extracted from national registries. All analyses were based on one-year age- and sex- specific figures. The risk for CVD increased with every age above 85 years although the rate of increase leveled off with age. Over time, the risk for CVD and MI decreased for all ages, and for stroke for ages up to 89 years. However, the risk of MI increased until around 2001 in all age groups and both sexes but decreased after that. The overall mortality improved for all outcomes over the period 1994 to 2010, so did the survival within 28 days from an event. The average annual decline in mortality over all ages, 85 and above was 3% for MI, 2% for stroke and for 2% CVD. Corresponding figures for ages 60–84 was 4% for each of MI, stroke and CVD. The results were similar for men and women.

**Conclusions:**

Improvements in CVD risks observed among ages up to 85 years appear to have extended also to ages above 85 years, even if the rate of improvement plateaued with age. The improvements in survival for all ages up to 99 years give no support to the hypothesis that more fragile individuals reach higher ages. Additional research is needed to find out if improvement in survival can be seen also for the second and third event of CVD, stroke and MI.

## Introduction

Cardiovascular diseases, CVD, are a major cause of death and account for a substantial amount of disease in Sweden and worldwide. However, the age-standardized rates in CVD, both disease and mortality, have declined in many countries since the mid-1960s, [1], [2], [3] a development that has been referred to as the fourth stage of the epidemiological transition [4]. The progress has been fueled by both a decrease in several important risk factors and by advances in medical care [5]. Despite the rapid increase in the number and proportion of old (age >85 years) individuals in the population, CVD trends for this group have not been presented in any detail. CVD trends have either been published exclusively for populations up to 85 years of age [6], [7], [8], or with the ages above 80 or 85 years as an open age group [9], [10], [11], or in one or two pooled age groups for the oldest [12], [13]. It is difficult to draw conclusions from CVD trends for an open age group of the oldest because the mean age will increase over time and, thus, age-related confounding may occur. Hence, it is not clear what the age specific CVD trends look like for those 85 years and above. This group is growing and because the incidence of CVD increases with age, it is of interest to see whether the observed downward trends in CVD among younger age-groups are also present in older age-groups. Given the improvement in life expectancy in old age [14], one can also expect to see decreases in CVD-associated mortality in older age-groups because this is the leading cause of death. On the other hand, improvements in CVD disease and mortality among younger individuals could potentially result in more fragile individuals (with increased risks compared to previous birth cohorts of the same age) reaching higher ages [15], [16]. This possibility is why a decrease in CVD-associated mortality among the oldest is not necessarily to be expected just because the risk decreased in younger age groups.

To obtain a comprehensive picture of the disease trends in a population it is necessary to examine different aspects together such as disease risks (incidence), separating first events from recurrent events, death risks among those with the disease (case fatality), and overall death risks [17]. Decreasing overall death risks might be the result of either decreasing disease risks or decreasing case fatality, or both. Decreasing risk of first CVD events is an indicator of the effect of primary prevention, while decreasing case fatality measures treatment effects. When interpreting decreasing mortality for the oldest it is essential to understand whether it is the reduced disease risks or improved survival that has been the driving the decrease. The latter may result in more years spent in ill-health, while reduced disease risks would lead to more years of healthy life.

The main aims of this study were to describe the age-specific trends in total CVD, as well as in myocardial infarction (MI) and stroke separately, among the oldest (85 to 99 years of age) in the population, with respect to incidence (first events), case fatality, and overall mortality. A further aim was to compare the trends among those below 85 years of age to those above 85 years of age.

## Methods

### Material

Sweden, together with the other Nordic countries, has a unique system of registers where individuals can be linked through their personal identification number. For the purpose of this study we used the Register of the Total Population, the Longitudinal Integration Database for Health Insurance and Labor Market Studies (Swedish acronym LISA), the National Inpatient Register, and the Cause of Death Register. The Register of the Total Population includes everyone registered in Sweden since 1968 and was used to identify the population. The LISA database (http://www.scb.se/default____2154.aspx) includes information about income, pensions and social transfers reported by The Swedish Social Insurance Agency. LISA was used in order to verify that every person included in the study was indeed alive and living in Sweden. Because LISA is updated yearly, the quality of the data about the oldest is better in this registry than in the Register of the Total Population and thus the risk of including individuals who left Sweden without being removed from the Register of the Total Population was minimized. The requirement for individuals in our study population to be identified in both registers reduced the total time at risk by 1.1%.

The National Inpatient Register includes everyone who experienced a hospital admission (inpatient care) and was used to collect information about CVD/MI/stroke events (together with the Cause of Death Registry). The register is of high quality and has provided nation-wide coverage since 1987; the coverage includes primary and secondary diagnoses assigned by physicians [18]. The Cause of Death Register was used to identify all deaths from CVD/MI/stroke. It includes all deaths with ICD diagnoses (International Classification of Diseases) for underlying and contributing causes of death, including deaths occurring outside of Sweden for individuals registered in Sweden. Both The Cause of Death Register and the National Inpatient Register are maintained by The National Board of Health and Welfare (http://www.socialstyrelsen.se/english). All databases used in this study were linked using the individuals’ personal identification numbers. The linkage was conducted by Statistics Sweden and the researchers received data without the personal identification number.

### Setting

We included everyone who was born from 1886 to 1925, registered in Sweden in 1987, and who could also be identified in the LISA database. All CVD, MI, and stroke events that were registered in the National Inpatient Register or in the Cause of Death Register between the years 1987 and 2010 were identified. The follow-up ended at whichever of the following dates occurred first; the date of death, the date of first hospitalization for any CVD, the date of emigration, or 31st December 2010.

The National Inpatient Register reached national coverage in 1987 and so complete information was not available for events before 1987. This study focused on first event, therefore a transition period had to be applied in order to ensure that we captured the first event. First, a period of 7 years was chosen because this has been used by the Swedish National Board of Health and Welfare in studies of incidence of MI [19]. Next, we tested whether the 7 year period was sufficient by calculating the incidence using the longest possible follow up period, 23 years (from 1987 to 2010), and by comparing the results to the incidence calculated after restriction to 7 years (from 2003–2010). Allowing only a 7-year transition period in order to identify a first event resulted in an overestimation of the incidence in the beginning of the period by approximately 10% depending on the diagnosis and age. To account for this, age- and diagnosis-specific underestimation correction factors, derived from the comparison, were applied for the years 1994 to 2010. After 2003, almost no effect was seen from the difference in follow up time. Therefore, the correction factors weighted the disease risks in the first years; the period when the follow up was too short.

We used the International Classification of Diseases (ICD) codes to identify the events. All CVD was defined as I00-I99-8 in ICD 10 and 390–459 ICD 8 and 9, MI as I21 and I22 in ICD10 or 410 in ICD 8 and 9, and stroke was defined as I60-I69 or G45 in ICD 10 or 430–438, 342 or 344 in ICD 8 and 9. Thus, all CVD included stroke and MI, as well as several other diagnoses. We analysed the trends based on underlying/main diagnose only and also based on all diagnoses including the contributing/secondary diagnoses. As expected, there was no difference in the trends for MI and stroke depending on whether only the underlying/main diagnosis or all diagnoses were used; even though the number of events was slightly reduced when we used the first diagnosis alone. All CVD, however, is a broad category and includes many milder and common diagnoses such as hypertension, occlusion and stenosis of cerebral arteries not resulting in cerebral infarction and atherosclerosis of other arteries that are set as secondary diagnoses. Therefore a larger difference was expected when using only the underlying/main diagnose or all secondary diagnoses. This was indeed the case; the incidence of all CVD was considerably higher when all diagnoses were included and the time trends increased. We found that it was more appropriate to present CVD trends based only on the underlying/main diagnosis. Because we wanted to be consistent, all trends are presented based on underlying/main diagnosis.

### Statistical Analyses

We computed age specific one-year disease- and death risks for the ages between 85 and 99 years of age. All new events in one calendar year were divided by the total number of persons at risk at the beginning of the year (the year they would turn 85, 86 and so on). Disease risks were based on all first events, fatal or non-fatal. Case fatality was calculated by dividing all deaths, regardless of diagnosis, within 28 days of an event by the number of events. Death risks were calculated as all deaths from CVD, MI or stroke divided by the population at risk each year. The analyses of CVD, MI, and stroke were run separately. All trends are presented as three year moving averages, and thus the figures presented are for the years 1995 to 2009 even though the analyses ran from 1994 to 2010.

The risk of cardiovascular disease increased with age. To estimate whether the risk increase was similar over the whole age range, we estimated the average relative risk increase with age over time in a discrete time logistic model [20]. In this regression model, the risk increase of one age was compared with that of the previous age; 86 vs. 85, 87 vs. 86 etc.

Finally, to estimate the average trends in disease and death risks, and in case fatality of all age groups over the entire time period, the same type of regression model was used. In this model younger ages were also included in order to compare the trends over time for different ages. The relative risk of an event per increase in calendar year was calculated in eight age groups, 60–64, 65–69, 70–74, 75–79, 80–84, 85–89, 90–94, 95–99, and for two combined age groups 60–84 and 85–99. With this strategy we could, in addition to the average effect for all ages above 85, see whether the period effect on the disease, mortality and case fatality were similar over age groups or if, for example, younger age-groups benefited more from decreasing CVD trends over time than older ones. The regression models were run for CVD, MI, and stroke. Statistical analyses were performed in Microsoft Excel and SAS version 9.2.

### Ethical Permission

An ethics approval for this study was obtained from the regional ethics committee in Stockholm, Dnr. 2011/136-31/5.

## Results

The calculations of death risks for all CVD (85 years and older) were based on 1,358,571 person years for men and 2,825,388 person-years for women.

### Age Specific CVD Trends

**Figure 1:**
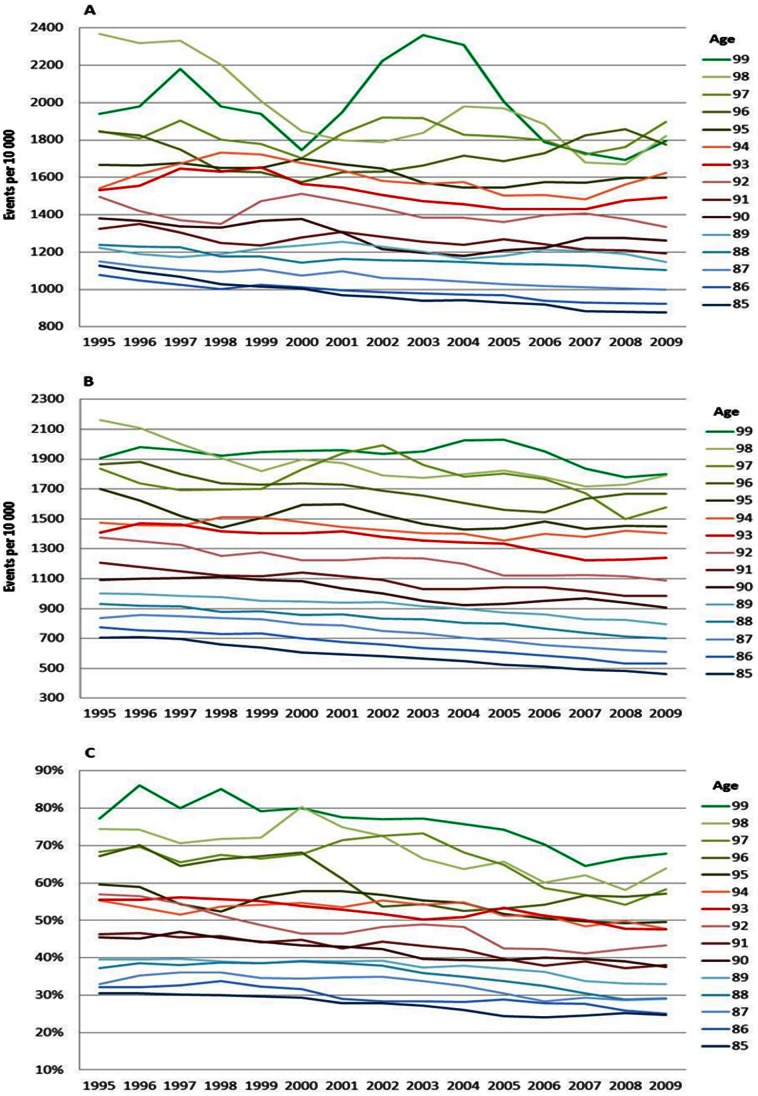
CVD incidence (A), mortality (B) and case fatality (C), men. Age specific trends in cardiovascular diseases, men, between 1994 and 2010. One year cumulative incidence (A), overall mortality (B), and, case fatality within 28 days, percent (C). Three year moving average.

**Figure 2:**
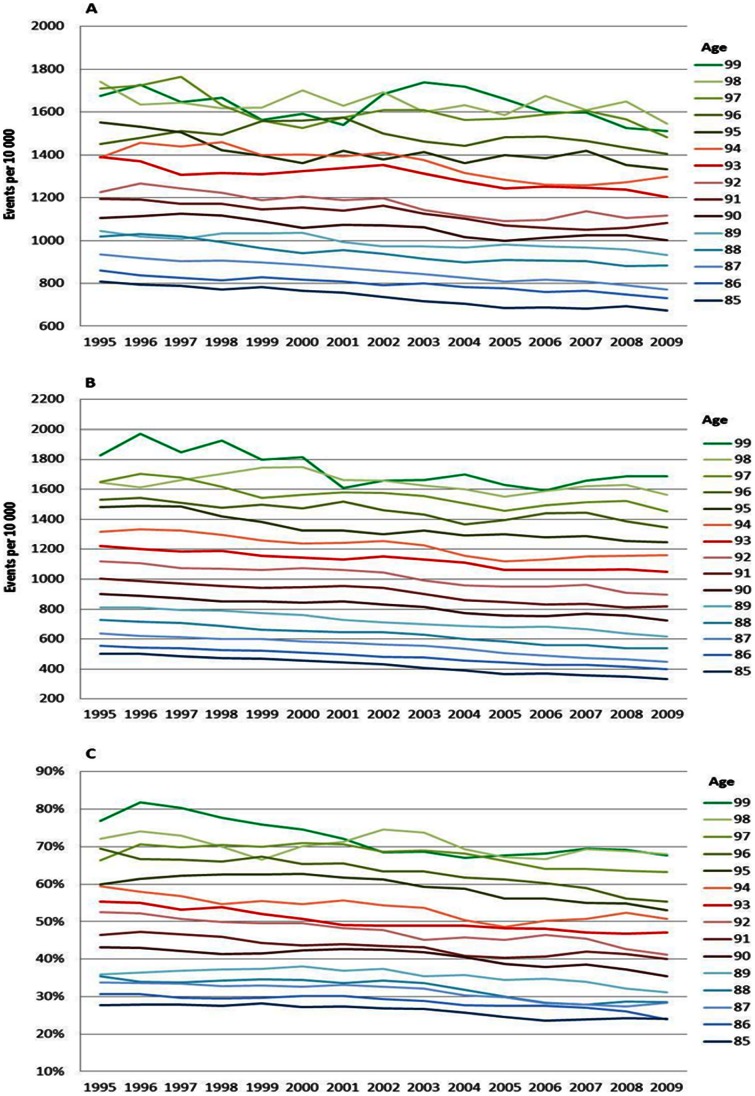
CVD incidence (A), mortality (B) and case fatality (C), women. Age specific trends in cardiovascular diseases, women, between 1994 and 2010. One year cumulative incidence (A), overall mortality (B), and, case fatality within 28 days, percent (C). Three year moving average.

The age specific CVD risks decreased between 1995 and 2009 for all ages and for both men and women (figures 1A and 2A). Nevertheless, the oldest male age-groups (aged 97–99 years) exhibited some fluctuations (figure 1A). The mortality and case fatality from CVD also declined over the entire period for men (figures 1B and 1C) and women (figures 2B and 2C) of all ages. The decline in overall mortality and case fatality was more pronounced than the decline in disease from CVD.

### Age Specific MI Trends

**Figure 3:**
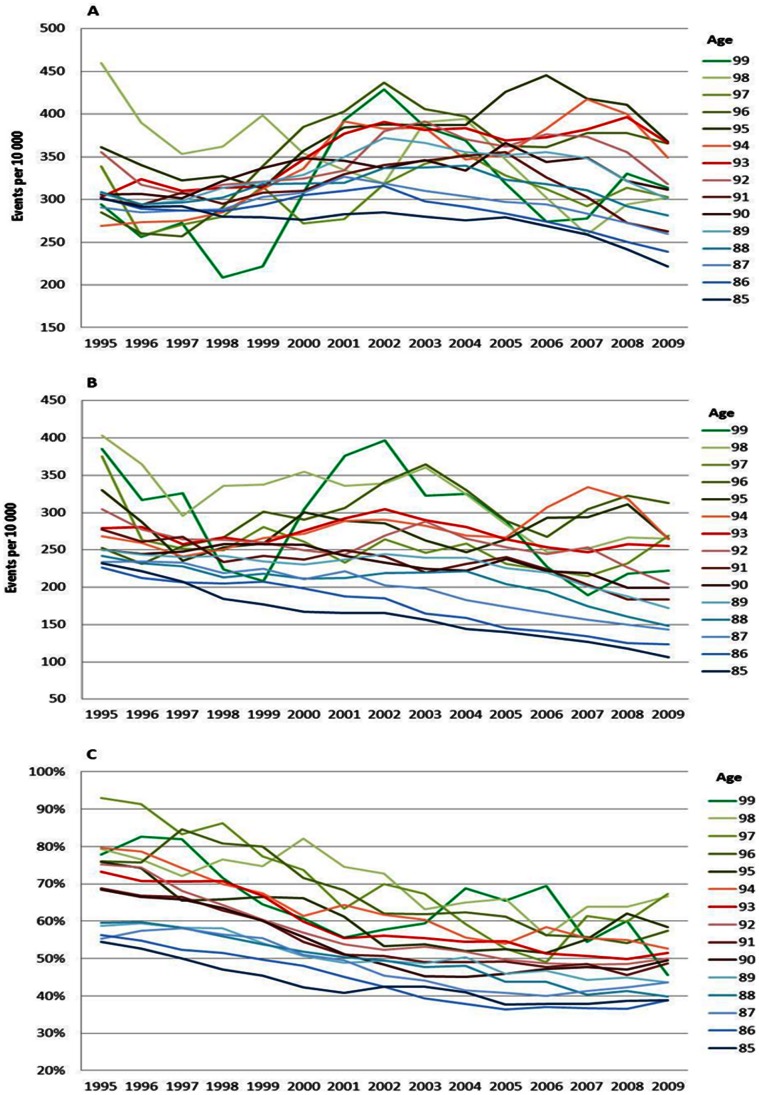
MI incidence (A), mortality (B) and case fatality (C), men. Age specific trends in myocardial infarction, men, between 1994 and 2010. One year cumulative incidence (A), overall mortality (B), and, case fatality within 28 days, percent (C). Three year moving average.

The MI trends showed larger fluctuations than the trends for all CVD due to fewer events, at least at the very highest ages, which made it more difficult to interpret the magnitude and even direction. However, the disease risks in MI showed a different pattern than the disease risks of all CVD. Instead of a continuous decline, the risks increased between 1995 and 2002, and then started to decrease (except for 85 year olds that did not increase). This pattern was seen for both men (figure 2A) and women (figure 3A) and in all ages except some ages in the higher 90s, where no decrease was seen. Death risks in MI declined between 1995 and 2009 for the ages up to 90, men (figure 2B) and women (figure 3B). Above age 90, the mortality risks remained stable. The differences between ages increased over time as a result of larger decreases in the younger age-groups than in the older ones. Case fatality showed a strong and similar decline for all ages between 1995 and 2009, men (figure 2C) and women (figure 3C). The improvement in case fatality was between 15 and 25% depending on age and gender.

### Age Specific Stroke Trends

For both men and women the risk of stroke did not decrease as much as the risk of CVD and MI between 1995 and 2009. Still there have been improvements, especially in the disease risks among men aged 85 to 89 years (figure 5A) and in overall mortality risks for all ages (figures 5B and 6B). For women, improvements were seen in disease risks between 70 and 89 years (figure 6A). Case fatality was stable for both men (figure 5C) and women (figure 6C) and showed less improvement than the overall death risks.

### Relative Risk Increase with Age

**Figure 4:**
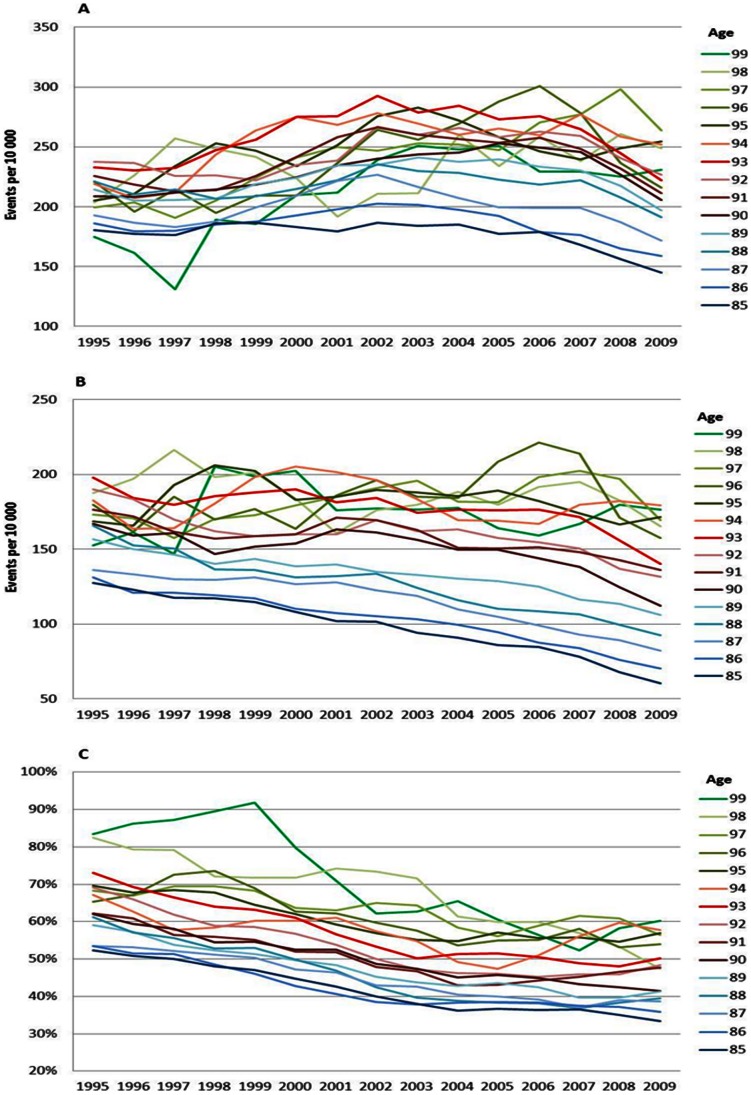
MI incidence (A), mortality (B) and case fatality (C), women. Age specific trends in myocardial infarction, women, between 1994 and 2010. One year cumulative incidence (A), overall mortality (B), and, case fatality within 28 days, percent (C). Three year moving average.

**Figure 5:**
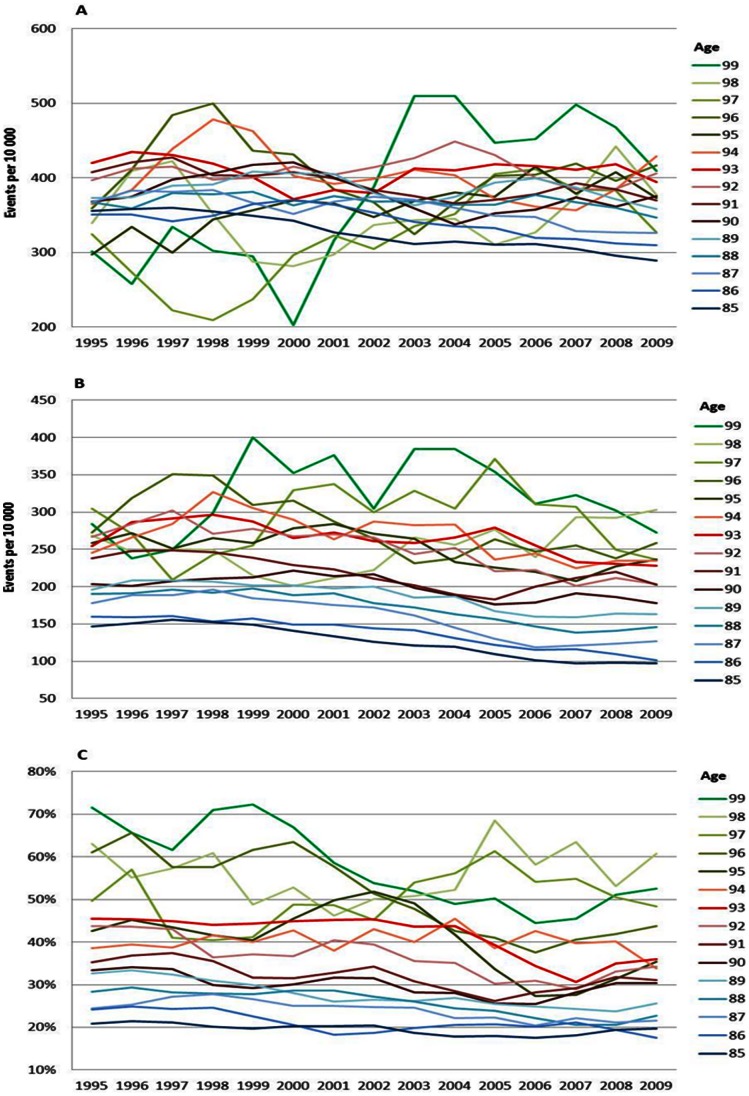
Stroke incidence (A), mortality (B) and case fatality (C), men. Age specific trends in stroke, men, between 1994 and 2010. One year cumulative incidence (A), overall mortality (B), and, case fatality within 28 days, percent (C). Three year moving average.

**Figure 6:**
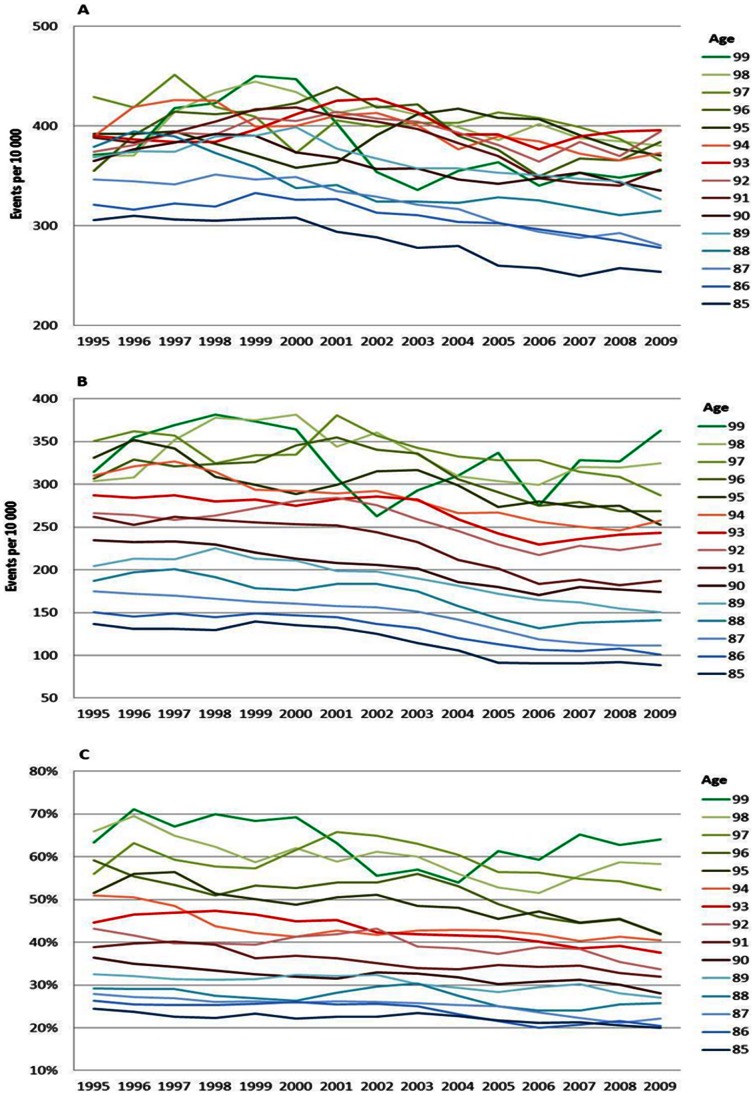
Stroke incidence (A), mortality (B) and case fatality (C), women. Age specific trends in stroke, women, between 1994 and 2010. One year cumulative incidence (A), overall mortality (B), and, case fatality within 28 days, percent (C). Three year moving average.

**Figure 7:**
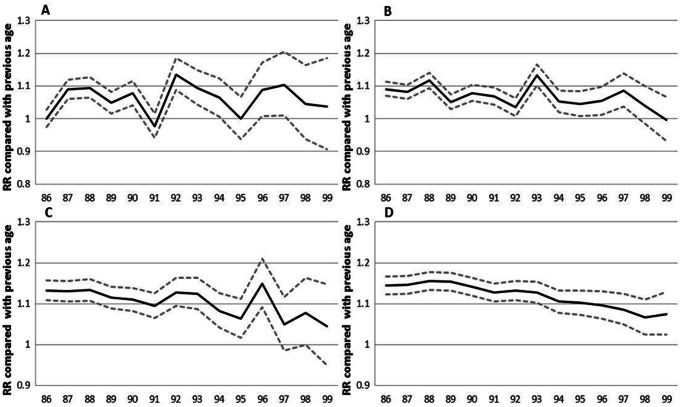
Relative risk of age on risk of CVD, men and women. Relative risk increase of age on risk of cardiovascular disease (men A, women B) and mortality from cardiovascular diseases (men C, women D). Solid line is relative risk, RR, and dashed lines upper and lower 95% confidence intervals. RR = 1 indicates no increased risk.

The increase in the risk of a CVD, MI and stroke event by age can be viewed in figures 1, 2, 3, 4, 5, and 6 by the parallel shift of the trend lines for successive higher age-groups. To find out whether the risk of CVD increased exponentially with age, we ran a regression model where each age was compared with the previous one. Figure 7A-D presents the relative risk increase of age for CVD disease and mortality for men and women. The relative risk for a CVD event per one year increase in age was about 9% (RR 1.09) for every successive year (for both men and women) between 85 to around 94 years; but, the increase leveled off and reached 3–4% at the highest ages (99 years; figure 7A and B). The fluctuations were larger for males than females. The relative risk of CVD death with age was 13% (RR 1.13) at age 86 compared with 85 for men. Again, the increase leveled with age; approximately 4% (RR 1.04) for 99 year-olds compared with 98 year-olds (figures 7C and 7D). For females the corresponding figures were 14% (RR 1.14) and 7% (RR 1.07). For MI the relative risk with age was smaller than overall CVD, but showed the same age-related pattern with age for women (data not shown). The estimates for the males fluctuated too much for us to draw any conclusions about the relative effect of age (data not shown). For stroke, the relative risk increase with age leveled off with age for women, whereas the relative risk with age was rather similar between 85 and 99 for males (data not shown).

### Age Adjusted Trends - Comparing Ages 85 and above with Ages below 85

#### CVD

Table 1 presents the age-adjusted trends from the regression model. The average relative effect with each subsequent calendar year over the period 1994 to 2010 is presented for eight age groups as well as for the ages 85 and above compared with 60–84 years. The average annual decrease in risk of CVD over all ages 85 and above for the period 1994 to 2010 was about 1% for both men and women. The corresponding figure for ages below 85 (60–84 years) was 2%. The death risk decreased slightly more over the same period with an average yearly decline of 2% for ages 85 and above, and 4% for ages 60 to 84. The average annual decline in case fatality for men and women was 2% for those aged 85 and above as well as for those in the age-range 60–84 years. In conclusion, improvements in CVD-associated morbidity and mortality observed for ages below 85 years appear to extend also into old age (85 and above), even though the rate of decline was smaller. This conclusion was also supported by the results from the combined five-year age groups (table 1). The effect in those 85 years of age and older was largest for the youngest group (85–89). The improvement in survival (case fatality) has however been of similar effect size for those below 85 as for those above.

**Table 1:** Average annual change in CVD between 1994 and 2010, ages 60–99 years, men and women.

	Incidence**		Mortality+			Case fatalitŷ	
Age	RR* (95% CI)		RR* (95% CI)			RR* (95% CI)	
	Men	Women	Men	Women	Men		Women
60–64	0.98 (0.98, 0.98)	0.98 (0.98, 0.98)	0.95 (0.95, 0.95)	0.96 (0.95, 0.96)	0.98 (0.98, 0.98)		0.99 (0.98, 0.99)
65–69	0.98 (0.98, 0.98)	0.98 (0.98, 0.98)	0.95 (0.95, 0.95)	0.96 (0.95, 0.96)	0.98 (0.97, 0.98)		0.98 (0.98, 0.99)
70–74	0.98 (0.98, 0.99)	0.98 (0.98, 0.98)	0.95 (0.95, 0.95)	0.95 (0.95, 0.96)	0.97 (0.97, 0.97)		0.98 (0.98, 0.98)
75–79	0.98 (0.98, 0.99)	0.98 (0.98, 0.99)	0.96 (0.95, 0.96)	0.96 (0.96, 0.96)	0.97 (0.97, 0.98)		0.98 (0.98, 0.98)
80–84	0.99 (0.99, 0.99)	0.98 (0.98, 0.99)	0.97 (0.96, 0.97)	0.96 (0.96, 0.96)	0.98 (0.97, 0.98)		0.98 (0.98, 0.99)
85–89	0.99 (0.99, 0.99)	0.99 (0.99, 0.99)	0.98 (0.97, 0.98)	0.98 (0.97, 0.98)	0.98 (0.98, 0.98)		0.98 (0.98, 0.99)
90–94	0.99 (0.99, 1.00)	0.99 (0.99, 0.99)	0.99 (0.98, 0.99)	0.98 (0.98, 0.98)	0.98 (0.97, 0.98)		0.98 (0.98, 0.99)
95–99	1.00 (0.99, 1.00)	0.99 (0.99, 1.00)	0.99 (0.99, 0.99)	0.99 (0.99, 0.99)	0.97 (0.96, 0.98)		0.98 (0.98, 0.99)
**60–84**	0.98 (0.98, 0.98)	0.98 (0.98, 0.98)	0.96 (0.96, 0.96)	0.96 (0.96, 0.96)	0.98 (0.97, 0.98)		0.98 (0.98, 0.98)
**85+**	0.99 (0.99, 0.99)	0.99 (0.99, 0.99)	0.98 (0.98, 0.98)	0.98 (0.98, 0.98)	0.98 (0.98, 0.98)		0.98 (0.98, 0.99)

* Relative risk (RR) per one year later calendar year; presented for eight age groups And two combined age groups. 60–84 and 85+. Realtive risk estimates with upper and lower 95% confidence intervals. RR = 1 indicates no increased risk. (**) One year risk of CVD event; (+) Overall mortality risk of CVD; (ˆ) 28 days case fatality of CVD.

### Myocardial Infarction

The average annual decrease in risk of MI over all ages 85 and above for the period 1994 to 2010 was stable (table 2). For those aged below 85 years, the average was 3% per annum. However, for those aged 85 years and above, the risk of MI increased in the beginning of the period and then decreased around 2001; thus, the net effect was no change. We therefore re-estimated the regression model starting from 2002 and isolated the period where the risk decreased. The average annual decline for both men and women was then 3% for those aged 85 years and older versus 4% for those aged 60–84 years. The death risk for MI decreased over the whole period; however, the decrease was greater for the younger (6%) than for the older (3%) cohort. Case fatality within 28 days of an MI improved for all ages; the average yearly decline was 5% for both men and women.

**Table 2:** Average annual change in MI between 1994 and 2010, ages 60–99 years, men and women.

	Incidence**		Mortality+		Case fatalitŷ	
Age	RR* (95% CI)		RR* (95% CI)		RR* (95% CI)	
	Men	Women	Men	Women	Men	Women
60–64	0.97 (0.97, 0.98)	0.98 (0.98, 0.98)	0.93 (0.93, 0.94)	0.94 (0.93, 0.95)	0.96 (0.95, 0.96)	0.96 (0.95, 0.97)
65–69	0.97 (0.97, 0.97)	0.98 (0.97, 0.98)	0.94 (0.93, 0.94)	0.94 (0.93, 0.94)	0.96 (0.95, 0.96)	0.96 (0.95, 0.96)
70–74	0.97 (0.97, 0.97)	0.98 (0.97, 0.98)	0.93 (0.93, 0.93)	0.93 (0.93, 0.94)	0.95 (0.95, 0.96)	0.95 (0.94, 0.95)
75–79	0.97 (0.97, 0.97)	0.98 (0.98, 0.98)	0.94 (0.93, 0.94)	0.94 (0.94, 0.94)	0.95 (0.95, 0.96)	0.95 (0.95, 0.96)
80–84	0.98 (0.98, 0.98)	0.98 (0.98, 0.98)	0.95 (0.95, 0.95)	0.95 (0.94, 0.95)	0.95 (0.96, 0.95)	0.95 (0.95, 0.95)
85–89	0.99 (0.99, 0.99)	0.99 (0.99, 1.00)	0.97 (0.96, 0.97)	0.97 (0.96, 0.97)	0.95 (0.95, 0.95)	0.95 (0.95, 0.95)
90–94	1.00 (1.00, 1.01)	1.00 (1.00, 1.01)	0.99 (0.98, 0.99)	0.98 (0.98, 0.99)	0.94 (0.93, 0.94)	0.95 (0.94, 0.95)
95–99	1.01 (1.00, 1.02)	1.01 (1.01, 1.02)	0.99 (0.98, 1.00)	0.99 (0.99, 1.00)	0.94 (0.92, 0.95)	0.94 (0.93, 0.95)
**60–84**	0.97 (0.97, 0.97)	0.98 (0.98, 0.98)	0.94 (0.94, 0.94)	0.94 (0.94, 0.94)	0.95 (0.95, 0.96)	0.95 (0.95, 0.95)
**85+**	1.00 (1.00, 1.00)	1.00 (1.00, 1.00)	0.97 (0.97, 0.98)	0.97 (0.97, 0.98)	0.95 (0.94, 0.95)	0.95 (0.94, 0.95)

* Relative risk (RR) per one year later calendar year; presented for eight age groups And two combined age groups. 60–84 and 85+. Realtive risk estimates with upper and lower 95% confidence intervals. RR = 1 indicates no increased risk. (**) One year risk of MI; (+) Overall mortality risk of MI; (ˆ) 28 days case fatality of MI.

### Stroke

The risk of stroke showed less improvement than the risk of all CVD and MI. For men, the relative risk per year for all ages above 85 was equal to 1; i.e., no age-related change (table 3). The corresponding figure for males aged between 60 and 84 years was 0.99; i.e., an average yearly decline of 1%. For women, there was an average yearly decline of 1% for both age-groups. The mortality risk decreased for both men and women aged 85 years and older; 2% per year. The corresponding figure for men and women in the 60–84 year-old cohort was 4%. Twenty-eight- day survival after stroke for men and women improved by an average of 2% per year for both age-groups.

**Table 3:** Average annual change in stroke between 1994 and 2010, ages 60–99 years, men and women.

	Incidence**		Mortality+		Case fatalitŷ	
Age	RR* (95% CI)		RR* (95% CI)		RR* (95% CI)	
	Men	Women	Men	Women	Men	Women
60–64	0.99 (0.99, 0.99)	1.00 (1.00, 1.00)	0.95 (0.95, 0.96)	0.96 (0.96, 0.97)	0.97 (0.96, 0.98)	0.97 (0.96, 0.98)
65–69	0.99 (0.99, 0.99)	1.00 (1.00, 1.00)	0.96 (0.95, 0.96)	0.96 (0.96, 0.97)	0.97 (0.97, 0.98)	0.97 (0.96, 0.98)
70–74	0.99 (0.99, 0.99)	0.99 (0.99, 0.99)	0.96 (0.96, 0.96)	0.95 (0.95, 0.96)	0.98 (0.97, 0.98)	0.97 (0.96, 0.98)
75–79	0.99 (0.98, 0.99)	0.99 (0.98, 0.99)	0.96 (0.96, 0.97)	0.96 (0.96, 0.96)	0.98 (0.98, 0.99)	0.98 (0.98, 0.99)
80–84	0.99 (0.98, 0.99)	0.98 (0.98, 0.99)	0.97 (0.97, 0.97)	0.96 (0.96, 0.97)	0.98 (0.98, 0.99)	0.98 (0.98, 0.99)
85–89	0.99 (0.99, 1.00)	0.99 (0.99, 0.99)	0.98 (0.97, 0.98)	0.97 (0.97, 0.98)	0.98 (0.98, 0.99)	0.99 (0.98, 0.99)
90–94	1.00 (1.00, 1.00)	1.00 (0.99, 1.00)	0.99 (0.98, 0.99)	0.98 (0.98, 0.98)	0.98 (0.97, 0.99)	0.98 (0.98, 0.99)
95–99	1.01 (1.00, 1.02)	1.00 (0.99, 1.00)	0.99 (0.98, 1.00)	0.99 (0.99, 0.99)	0.97 (0.96, 0.99)	0.98 (0.97, 0.99)
**60–84**	0.99 (0.99, 0.99)	0.99 (0.99, 0.99)	0.96 (0.96, 0.97)	0.96 (0.96, 0.96)	0.98 (0.98, 0.98)	0.98 (0.98, 0.98)
**85+**	1.00 (1.00, 1.00)	0.99 (0.99, 0.99)	0.98 (0.98, 0.98)	0.98 (0.98, 0.98)	0.98 (0.98, 0.98)	0.98 (0.98, 0.98)

* Relative risk (RR) per one year later calendar year; presented for eight age groups And two combined age groups. 60–84 and 85+. Realtive risk estimates with upper and lower 95% confidence intervals. RR = 1 indicates no increased risk. (**) One year risk of stroke; (+) Overall mortality risk of stroke; (ˆ) 28 days case fatality of stroke.

In conclusion, people aged between 60–84 years experienced a greater reduction with respect to disease and death risks than those aged 85 years and older. In contrast, the improvement in case fatality was similar in both cohorts.

## Discussion

We determined age-specific trends for disease risk, death risk, and 28 day-case fatality for CVD, MI and stroke for men and women aged 85 to 99 years in Sweden for the years 1994 to 2010 using nationwide registry data. For comparison, age-adjusted trends for the ages 60–84 were also calculated. To our knowledge, no other study has presented age-specific cardiovascular trends for such an old cohort. The results revealed that improvements in morbidity and mortality were evident for the oldest age-groups, with a few exceptions (MI incidence in the beginning of the period and stroke risk among the very oldest, 90–99 years). In a previous study, we calculated how much the incidence would need to decrease in order to compensate for the demographic changes; primarily changes in the age structure [21]. We concluded that even though there will be substantial demographic changes in the coming decades, the absolute number of stroke and MI events will not necessarily increase because of the downward trends in disease and mortality from stroke and MI. In that paper, our calculations were based on the entire Swedish population aged 20 years and older. The results of the present study, focusing on the oldest old, support our earlier conclusion, even if the improvements in stroke risk may not completely meet the required decline. However, even if both disease risks and mortality risks decline, the larger improvement in survival versus disease risk suggests that older individuals may spend more years in ill-health. We should note that in the current study we were only able to assess people younger than 100 years of age. In a previous article, we found that although there was a decline in mortality up to 100 years of age, this decline was not found in older subjects [22]. Thus, it is possible that the positive trends found in this paper do not apply to centenarians.

### Comparison with Other Studies

Both disease- and death risks from CVD, MI, and stroke increased with age, but the rate of increase reached a plateau. This is in accord with an American study of CVD showing that the rate of increase with age seemed to level off at the highest ages [23] and consistent with our own previous studies [22], [24]. CVD has, in some studies, been investigated in terms of non-fatal events and a plateau has been observed around the age of 90 years [23], [25]. This is probably because increased risk is outpaced by the competing risk of death at the highest ages [23]. In our study, we defined incidence of CVD as the first event (either fatal or non-fatal), and that is probably why we did not observe a plateau. We did, however, observe a decrease in CVD incidence over the whole period for all ages.

Our general finding of decreasing trends in CVD over time also for the oldest is also consistent with the few others studies that have addressed this issue; although such studies usually examined aggregated age groups. For example, Vaartjes and colleagues found decreasing trends for mortality from coronary heart disease [26], and stroke [13] for the age group 85–94 years in the Netherlands. Furthermore, they found the stroke incidence to be stable and the case fatality improved. Again, this is consistent with our results, even if their rates for case fatality were lower [13]. Another study by Koopman and colleagues looked at trends in acute MI (AMI) and found that both incidence and case fatality in AMI decreased between 1997 and 2008 in the Netherlands [12]. In our study, the incidence of MI increased until around 2002 and then started to decrease. We have no explanation for this, but others have reported similar findings [27]. Koopman and colleagues also observed the largest declines in MI incidence from 2003 onwards [12]. Regarding the larger decline in mortality than morbidity in our study, the same finding was noted in an earlier study by Amiri and colleagues where they noted that mortality from ischemic heart disease (IHD) had fallen more than morbidity from IHD [11]. They concluded that therefore the priority of prevention and treatment of IHD will have to be shifted from prevention of death to prevention of disability.

Although men had higher absolute risk for all three outcomes in our study, the time trends were very similar in men and women for disease, mortality and case fatality. These results are in line with a Norwegian study presenting similar incidence and case fatality trends for myocardial infarction in old age for men and women [28]. Hollander and colleagues [8] reported the patterns and life-time risk of stroke for older men and women to be similar; even though men had a higher incidence rates at all ages [8].

### Strengths and Limitations

An important issue to consider when studying disease trends for the oldest is the diagnostic data quality. The diagnoses for disease and mortality data are likely to be of lower quality for the very old compared with younger patients because of co-morbidity. For example it may be difficult to separate a main diagnose from a secondary diagnose. However, it is likely that the quality is worse in specific diagnoses rather than in broad ones, such as all CVD. For MI and stroke, it is possible that both specificity (were events missed?) and sensitivity (was the disease misclassified such that individuals were classified with CVD, MI or stroke when they in fact had another outcome?) are problems. In 2001, new diagnostic criteria for in-hospital MI were introduced. The National Board of Health and Welfare in Sweden, the agency responsible for the statistics of inpatient care and causes of death, reported an increase between the years of 2000 and 2003. They attributed this increase to the new diagnostic criteria; after this initial increase, the incidence then decreased. In terms of the specificity, we examined the effect of including both underlying and contributing causes of death as well as only the main cause. As mentioned in the methods, there was no effect on the trends with the exception of incidence in all CVD. For the sensitivity aspect, we examined the number of events that had not been examined at hospital before death or those that had been confirmed at autopsy; because these could be considered unconfirmed events. For MI, this amount was around 60% in the beginning of the period and has increased steadily to reach almost 90% at the end of the period. The number of autopsies performed has not increased, but the probability of having an examination at hospital before death has. The National Board of Health and Welfare in Sweden reported that among those above 75 years, the proportion of autopsies decreased from 38 percent in 1970 to 7 percent in 2005 (Swedish National Board of Health and Welfare, available at http://www.socialstyrelsen.se/publikationer2007/2007-42-15/Summary accessed 2013 April 19th). However, the techniques of assigning diagnoses (for example, the use of CT scans and more sensitive troponin assays) have improved since 1970 and so the autopsy rate alone cannot be considered as a single valid predictor of the quality of the causes of death data.

Regarding examination at hospital before death, there was an apparent discrepancy with a smaller proportion of unconfirmed events among the younger group and a larger proportion among the oldest in 1995–2000. At the end of the study period, 2005–2010, the proportion of unconfirmed events was only around 10% and there was no age-related difference. Therefore, it is possible that the disease risks were over-estimated for the oldest (if sensitivity is the assumed problem). However, because we applied correction factors, we automatically made our definition of events more conservative. This occurs because the weighting used in the correction factors derives from information about the first and subsequent events applied backwards starting from 2010.

### Conclusions

Improvements in CVD-related morbidity and mortality observed among people aged up to 85 years in the past several decades appear to extend also to those aged 85 years and older even though the rate of improvement leveled off with age. At the same time, the improvements in 28 days survival after a CVD-associated first event for all outcomes and all ages does not support the suggestion that more fragile individuals reach advanced age. Our findings of downward trends in CVD, including MI, suggest that the challenges from CVD posed by an ageing population may not be as severe as they might appear when considering the demographic component alone. Additional research is needed to determine how improved survival affects the number of events at higher ages and also determine if improvement in survival occurs also after the second and third events.
